# Blood-Based Biomarkers of Aggressive Prostate Cancer

**DOI:** 10.1371/journal.pone.0045802

**Published:** 2012-09-28

**Authors:** Men Long Liong, Chun Ren Lim, Hengxuan Yang, Samuel Chao, Chin Wei Bong, Wing Seng Leong, Prashanta Kumar Das, Chit Sin Loh, Ban Eng Lau, Choon Geok Yu, Edie Jian Jiek Ooi, Robert K. Nam, Paul D. Allen, Graeme S. Steele, Karl Wassmann, Jerome P. Richie, Choong Chin Liew

**Affiliations:** 1 Lam Wah Ee Hospital, Georgetown, Penang, Malaysia; 2 GeneNews (Malaysia), Mount Miriam Cancer Hospital, Penang, Malaysia; 3 GeneNews Ltd, Richmond Hill, Ontario, Canada; 4 Gleneagles Intan Medical Centre, Kuala Lumpur, Malaysia; 5 Loh Guan Lye Hospital, Penang, Malaysia; 6 Sunnybrook Health Sciences Centre,Toronto, Ontario, Canada; 7 Brigham and Women's Hospital, Harvard Medical School, Boston, Massachusetts, United States of America; The Chinese University of Hong Kong, Hong Kong

## Abstract

**Purpose:**

Prostate cancer is a bimodal disease with aggressive and indolent forms. Current prostate-specific-antigen testing and digital rectal examination screening provide ambiguous results leading to both under-and over-treatment. Accurate, consistent diagnosis is crucial to risk-stratify patients and facilitate clinical decision making as to treatment versus active surveillance. Diagnosis is currently achieved by needle biopsy, a painful procedure. Thus, there is a clinical need for a minimally-invasive test to determine prostate cancer aggressiveness. A blood sample to predict Gleason score, which is known to reflect aggressiveness of the cancer, could serve as such a test.

**Materials and Methods:**

Blood mRNA was isolated from North American and Malaysian prostate cancer patients/controls. Microarray analysis was conducted utilizing the Affymetrix U133 plus 2·0 platform. Expression profiles from 255 patients/controls generated 85 candidate biomarkers. Following quantitative real-time PCR (qRT-PCR) analysis, ten disease-associated biomarkers remained for paired statistical analysis and normalization.

**Results:**

Microarray analysis was conducted to identify 85 genes differentially expressed between aggressive prostate cancer (Gleason score ≥8) and controls. Expression of these genes was qRT-PCR verified. Statistical analysis yielded a final seven-gene panel evaluated as six gene-ratio duplexes. This molecular signature predicted as aggressive (ie, Gleason score ≥8) 55% of G6 samples, 49% of G7(3+4), 79% of G7(4+3) and 83% of G8-10, while rejecting 98% of controls.

**Conclusion:**

In this study, we have developed a novel, blood-based biomarker panel which can be used as the basis of a simple blood test to identify men with aggressive prostate cancer and thereby reduce the overdiagnosis and overtreatment that currently results from diagnosis using PSA alone. We discuss possible clinical uses of the panel to identify men more likely to benefit from biopsy and immediate therapy versus those more suited to an “active surveillance” strategy.

## Introduction

Prostate cancer is the most common form of cancer in men in the United States (after skin cancer) [1]. Screening for the disease is usually by digital rectal examination (DRE) and prostate-specific antigen (PSA) assay. However, the benefits of PSA/DRE screening are controversial, due to the high false positive rate, low positive predictive value (PPV) and reported poor accuracy in identifying men affected by aggressive prostate cancer. In the past few years, two large prospective trials from the United States and Europe have highlighted the ambiguity in the value of PSA-based methods for prostate cancer screening. The Prostate, Lung, Colon and Ovarian Cancer Screening Trial (PLCO) in the U.S. enrolled more than 76,000 men randomized to receive yearly PSA screening for six years and DRE for four years versus “usual care” [2]. The authors found no difference in carcinoma of prostate (CaP)-related mortality between the two groups. The European Randomized Study of Screening for CaP (ERSPC) Trial included 182,000 men between the ages of 50–74 years [3]. Although the authors reported a 20% reduction in CaP mortality in men who underwent PSA testing at least once every four years, this mortality reduction proved costly – for every one CaP death prevented, 1,410 men needed to be screened annually and 48 men needed treatment. These trials have reinvigorated the debate over the utility and limitations of PSA screening [4]–[10].

Major problems in PSA testing arise as a result of over- and under-diagnosis. Some 15% of men whose PSA levels are regarded as normal (4·0 ng/mL or less), do in fact harbour prostate cancer, including high-grade carcinoma [9]. By increasing the limit to a level considered clinically borderline (4·0 –10·0 ng/mL), some 25% of men are found to be affected by prostate cancer [2]. Conversely, high PSA levels are observed in many men with indolent cancers [11]. It is estimated that overtreatment may occur in 40% to 50% of cases. Furthermore, many non-malignant conditions may affect PSA, including benign prostate enlargement and prostatitis. Confirmation of diagnosis requires some 12–18 core biopsies, at considerable cost and morbidity [12]. In light of these PSA-related challenges, it is evident that there is need for more clinically relevant biomarkers that are able to accurately predict the presence of aggressive prostate cancer.

A recent retrospective study identified a tissue-based mRNA expression signature of Gleason grade for predicting lethal prostate cancer [13]. Similar biomarkers, but blood-based and capable of identifying high-grade prostate cancer [Gleason score 7(4+3)–10] at an early stage (prior to decision on biopsy), would be clinically useful [14], [15]. Such a technique would complement current methodologies and help increase confidence in prostate cancer diagnosis and management.

In earlier work, we developed a novel blood-based biomarker panel able to risk- stratify patients for colorectal cancer [16]. The results of this test enable clinicians to encourage with greater confidence those patients with “high current risk“ scores to proceed with colonoscopy. Here we identify novel blood-based biomarkers for high-grade [Gleason score 7(4+3)–10] prostate cancer that can be used to risk-stratify PSA-positive men. This test could be useful to identify the subgroup of men for whom the benefit of biopsy is likely to outweigh the associated risk and discomfort.

Men with clinically localized low grade tumors are often advised to undergo surveillance rather than active treatment for a disease that is unlikely to progress. However, Borocas and colleagues have shown that fewer than 10% of men prefer surveillance over active treatment [17]. One suggested explanation for this is that even in low-risk men, the fear exists that a life-threatening, high grade cancer might be missed [18], [19]. A test that can predict the presence of potential high grade tumors might relieve patient anxiety, resulting in higher tolerance for surveillance.

## Materials and Methods

### Patients and blood samples

Sample acquisition for biomarker identification was conducted between 2004 and 2009 in urology clinics across North America (Toronto, Ontario, Canada and Boston, MA, USA) and Asia (Penang, Malaysia). Written, informed consent was obtained from all participants and approved by each institution's Research Ethics Board [Partners Health Care Institutional Review Board (Brigham and Women's Hospital, Boston), University Health Network Research Ethics Board (Sunnybrook Hospital, Toronto), and Joint Ethics Committee of School of Pharmaceutical Sciences (USM Hospital Lam Wah Ee on Clinical Studies (Penang)].

All blood samples were taken prior to any treatment or biopsies. Diagnosis was confirmed by transrectal ultrasound guided tru-cut® needle biopsy and/or by histopathological findings in radical prostatectomy specimens (See Appendix S1). When a sample had a radical prostatectomy report, the ultimate grade was based on the prostatectomy report. The number of core samples taken at biopsy was 6+6 for prostate volume <40 grams and 7+7 for volume >40 grams. Each institution's in-house pathologists determined diagnosis and evaluation of Gleason score. Cases with Gleason score  = 7 were excluded from the training dataset to control for gene expression differences of intermediate grade prostate cancer with Gleason score 7(3+4) which, we hypothesized, could be significantly different than more aggressive Gleason score 7(4+3) [14], [15]. Samples having Gleason score ≥ 8 at biopsy with or without prostatectomy were specifically selected for analysis and development of the gene signature with the training dataset. The model developed with the Gleason score ≥ 8 was then later applied to the patients with Gleason score 7 to determine if the molecular signature could differentiate between 7(3+4) versus more aggressive 7(4+3) Gleason scores.

In total, we collected 1,938 samples including 739 Prostate Cancer (PrCa) cases and 1,199 controls (G0). From this group, a 255 sample subset (n = 91 PrCa with Gleason score ≥ 8 and n = 164 controls) collected in EDTA tubes was set aside in an initial study for biomarker discovery on a microarray platform. Samples in each group were matched for age, race, BMI, and comorbidities (Table 1).

**Table 1:** Clinical characteristics of the patient cohorts for microarray hybridization.

North American Site				Asian Site			
**Characteristics**	**Non-cancer (G0)**	**Prostate Cancer (G8)**	P value*	**Characteristics**	**Non-cancer (G0)**	**Prostate Cancer (G8)**	P value*
**No. of Samples**	124	42		**No. of Samples**	40	49	
**Age (median)**	67	71	0.23	**Age (median)**	65.5	73	0.0001
**BMI (median)**	26.24	26.6	0.53	**BMI (median)**	NA	NA	
**Family History (%)**	13 (10.5%)	4(9.5%)	1.00	**Family History (%)**	0	0	1.00
**Ethnicity [no. (%)]**				**Ethnicity [no. (%)]**			
White	104 (83.9)	32 (76.2)	0.26	Chinese	29 (72.5%)	35 (71.4%)	1.00
Asian	11 (8.9)	5 (11.9)	0.38	Indonesian	4 (10.0%)	5 (10.2%)	1.00

**Figure 1:**
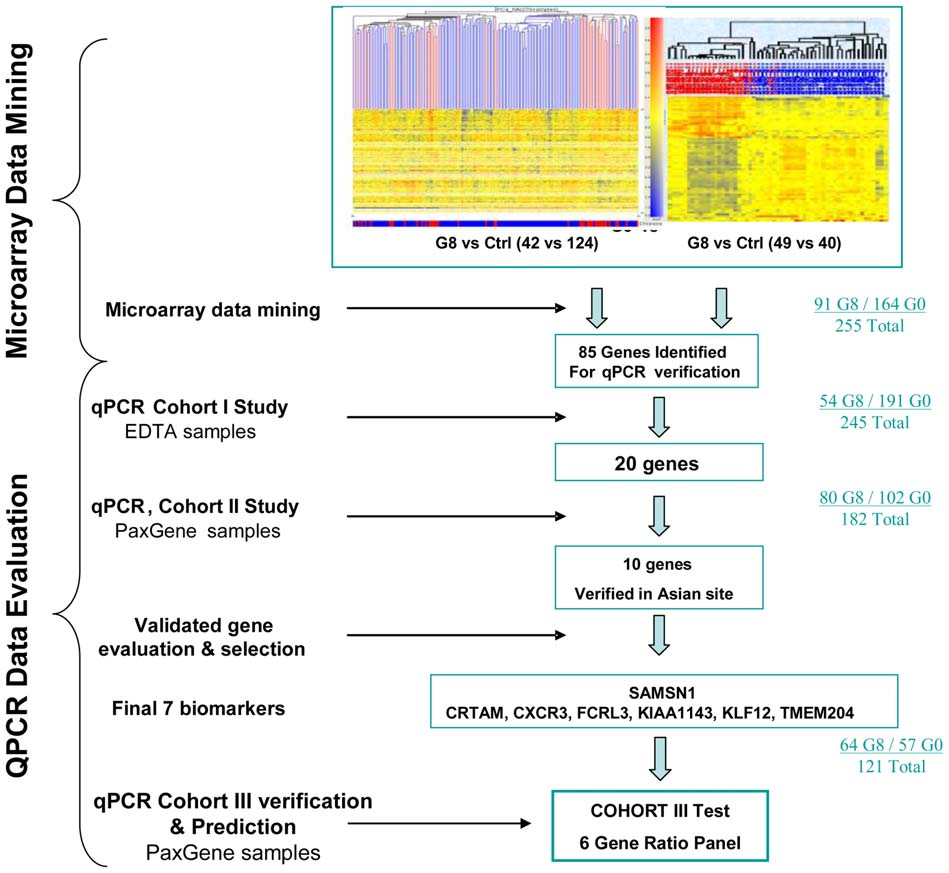
Gene identification and validation process. Gene Identification using Affymetrix U133Plus 2.0 GeneChip oligonucleotide arrays was carried out in Toronto, Canada, and Penang, Malaysia, in parallel. In Toronto, analysis was conducted on 166 samples (G8 = 42, G0 = 124). At the Malaysian site, 89 samples were profiled (49 G8, 40 G0). From microarray data analysis, 85 genes identified at both sites were tested in a series of quantitative real-time PCR verification studies. Twenty genes were verified through a Cohort I study on several cohorts of EDTA samples (total 245). These 20 genes were further tested in a Cohort II series of experiments on PAXgene samples (total 182), executed independently in Penang, Malaysia. 10 of the genes were verified, of which 7 genes became our final biomarkers and also confirmed in another independent sample set-Cohort III test (total 121).

For qRT-PCR verification studies, we tested the identified genes on most of the same samples hybridized for gene identification. We used 245 samples (G8 = 54, G0 = 191) for our Cohort I study, 182 samples (G8 n = 80 and controls n = 102) for our Cohort II study and 121 samples (G8 n = 64 and controls n = 57) as an independent test set for our Cohort III study (Figure 1). A Cohort IV group of cases with intermediate grade cancer (G6 n = 33, G7(3+4) n = 35, and G7(4+3) n = 43) was used to evaluate cancer aggressiveness performance.

Samples were excluded from analysis if an individual was determined to have had: 1) previous PrCa; 2) precancerous lesions, such as high-grade prostate intraepithelial neoplasm and atypical small acinar proliferation; 3) history of any cancer; and/or 4) severe medical conditions precluding treatment for prostate cancer, such as heart or renal failure.

Patients were divided into subgroups based on Gleason scores: G6 (Gleason score ≤ 6); G7 (Gleason score  = 7); G8 (Gleason score ≥ 8); and controls (G0). The North American control group comprised urology clinic patients with a single negative biopsy. The Malaysian control group consisted of men aged 50–75 years, negative for DRE, and whose PSA levels were below 2·5 ng/ml for five consecutive years, up to and including the date of recruitment. This extra condition effectively aligns the Malaysian controls with the North American yearly PSA screening standard. This also allowed us to ensure that PSA velocity remained below 0·4 ng/ml/yr, reducing the probability of missed carcinomas to a negligible value.

### Blood collection and RNA isolation

For microarray analysis and Cohort I, samples of peripheral whole blood (2×10 ml) were collected in EDTA Vacutainer® tubes (Becton Dickinson) (to avoid the high globin transcript problem on Affymetrix microarrays associated with the PAXgene system), and processed as described previously [20].

Samples were run on the Affymetrix U133 Plus 2.0 platform. Confirmational analysis was conducted using qRT-PCR for Cohort II and Cohort III. For qRT-PCR, blood collected in PAXgene™ tubes (PreAnalytiX) was processed according to PAXgene™ Blood RNA Kit protocol. The PAXgene system is more suitable for RT-PCR studies, and is a better fit for real-life clinical applications, due to its ability to immediately stabilize RNA and to keep it stable over a longer period of time, thereby providing greater flexibility in sample collection and transport.

RNA integrity was assessed using the 2100 Bioanalyzer RNA 6000 Nano Chip (Agilent Technologies). All samples met the following quality criteria: RIN≥7·0; 28S:18S rRNA ratio ≥ 1·0; and a validated Agilent bioanalyzer scan. RNA quantity was determined by absorbance at 260 nm in a DU-640 Spectrophotometer (Beckman Coulter).

### Microarray hybridization and data analysis

Microarray data mining to identify aggressive prostate cancer biomarkers involved two analyses simultaneously carried out at our North American (Toronto, Canada) and Asian sites (Penang, Malaysia). At the North American site, we hybridized 166 samples (G8 n = 42; G0 n = 124). Genes identified as significant (p<0.05, fold change ≥1.0, Benjamini-Hochberg FDR corrected) were selected for further downstream study using annotations and probe design, [available at the Affymetrix website (http://www.affymetrix.com)] and information on gene structure and function [available at National Institutes of Health website (http://www.ncbi.nlm.nih.gov)]. Similar profiling and data mining processes were undertaken at our Asian site in Malaysia with 89 samples (G8 n = 49; G0 n = 40), see Figure 1.

Microarray data files for the combined 91 G8 and 164 G0 samples were background corrected via GCRMA and imported into GeneSpring software (version 7·3·1, Agilent, California, USA) for analysis. Unreliable signals, as defined by the cross-gene error model, were discarded from analysis.

Probe set signal intensities were compared between disease and control groups. Genes were determined to be differentially expressed between cases and controls using the Analysis of Variance (ANOVA) test incorporated in GeneSpring. Probesets with p-values less than 0·05 and fold change magnitudes greater than 1·2 were identified as statistically significant. Sample size was determined based on an estimation we had made for a previous study, that is, that 100 samples per group are required to achieve adequate power (0.80), with a type I error of less than 0.05 and a fold change magnitude greater than 1.2, for a large proportion (over 75%) of genes being investigated [20].

Microarray data were also processed by MAS5. Probe sets marked as “absent” or “marginal” in any sample were discarded. The analysis was performed independently using the R program provided by Bioconductor.org (Seattle, Washington, USA). Only genes that appeared statistically significant in both collection sites were selected.

### Quantitative real-time polymerase chain reaction

Genes selected from the microarray analyses were verified using qRT-PCR in a Cohort I study. Only genes identified in microarray analysis that also remained statistically significant in the Cohort I qRT-PCR study were retained for further downstream analysis.

cDNA template for qRT-PCR was reverse-transcribed from RNA using the High Capacity cDNA Reverse Transcription Kit (Applied Biosystems). 20 ng of cDNA were used in a 25 µL reaction volume in a TaqMan® Duplex Reaction (for an overview of the methodology, see Figure 1).

Genes verified in Cohort I were then tested in a Cohort II study. Cohort II blood samples were collected in PAXgene™ tubes (PreAnalytiX), otherwise Cohort I and II study methodologies were identical.

The experiments were performed in duplex qRT-PCR tests. Each gene of interest was assayed in a series of experiments, with an endogenous reference gene (ACTB; beta actin). Baseline expression of ACTB allowed us to identify significantly differing levels of gene transcripts between the prostate cancer and control groups. Genes verified by both cohort analyses were combined as pairs; ratios of an overexpressed gene and an underexpressed gene were directly measured in duplex reactions. Gene expression differences were estimated using the comparative cycle threshold (Ct) method of relative quantification [21], normalizing the Ct values relative to the reference gene. This was performed by calculating a ΔCt_sample_  =  Ct_(target gene)_ – Ct_(partner gene)_. The relative fold-change (disease versus control) was represented as 2^−ΔΔCt^, where ΔΔCt  =  mean ΔCt_Ca_ – mean ΔCt_control_.

We chose SAMSN1, an overexpressed gene, as the partner gene with each of the six downregulated target genes. This format allows calculation of an “UP/DOWN” gene expression ratio between each underexpressed PrCa biomarker gene and its duplex partner, SAMSN1, from the difference of their Ct values as described previously [16]. A nonparametric Mann-Whitney test evaluated statistical significance of differences between control and PrCa mRNA levels.

## Results

From Cohort I (combined North American and Malaysian study sites) 85 genes were identified to be significantly differentially expressed between Gleason score ≥8 prostate cancer and controls, and selected for further investigation via qRT-PCR (Figure 1).

### Quantitative real-time PCR verification

These 85 genes were tested on 245 Cohort I samples collected in EDTA tubes (G8 n = 54; G0 n = 191). Twenty of the genes were verified as remaining significant in a qRT-PCR assay and retained for the Cohort II study.

Of the 20 candidate genes, ten remained significant in Cohort II samples collected in Paxgene tubes (p-value < 0.001, fold changes from 1·31 to 1·58 for overexpressed genes and -1·28 to -1·48 for underexpressed genes).

The expression values of the ten genes were analyzed using a multivariate logistic regression model. The AUC ROC of each single gene ranged from 0·60 to 0·69; paired comparison resulted in seven-, eight- or nine-gene combinations that all achieved an AUC of about 0·82. Of interest, the best seven-gene combination was comprised of one overexpressed gene and six underexpressed genes (AUC = 0·82). Thus we selected this seven-gene biomarker panel for detecting high-grade prostate cancer (Table 2).

**Table 2:** High grade prostate cancer (Gleason score 8 and above) biomarker gene list and differential expression ratio in Cohort II verification sample set (80 disease and 102 controls).

Gene Name#	Description	Sequence Accession ID	Expression Fold Change†	Expression P Value‡	Expression AUC*
CRTAM	cytotoxic and regulatory T cell molecule	NM_019604	1.58	3.46E-05	0.67
CXCR3	chemokine (C-X-C motif) receptor 3	NM_001504	1.59	3.38E-05	0.66
FCRL3	Fc receptor-like 3	NM_052939	1.61	2.85E-06	0.69
KIAA1143	KIAA1143	NM_020696	1.44	1.82E-07	0.73
KLF12	Kruppel-like factor 12	NM_007249; NM_016285	1.66	8.16E-07	0.71
TMEM204	transmembrane protein 204	NM_024600	1.52	8.40E-05	0.67
SAMSN1	SAM domain, SH3 domain and nuclear localization signals 1	NM_022136	–	–	–

# The 7 biomarkers were picked up from the 10 that were verified in Cohort II samples, using gene-ratio algorithm, based on the best AUC of combined gene-pair.

† Determined by qRT-PCR analysis using SAMSN1 as a partner gene, gene ratio was calculated using delta delta Ct calculation.

‡ Calculated by Mann-Whitney test.

* area under receiver-operating-characteristic curve.

We used the single overexpressed gene, SAMSN1, to be the common partner gene for each of the six underexpressed genes: CRTAM, CXCR3, FCRL3, KIAA1143, KLF12 and TMEM204. The six duplexes representing six gene expression ratios were evaluated on the same Cohort II samples (G8, n = 80; Ctrl, n = 102). The expression levels of the six duplexes were observed to differ significantly between disease and control groups (p<0.0001 and fold changes of 1·44–1·66; Table 2). Discriminative performance was also evaluated using AUC ROC The discriminative ability of the six duplex panel (combinative performance of the six duplexes) achieved an AUC of 0·74 (95%CI: 0·67–0·80), specificity of 84%, sensitivity of 63% and accuracy of 75%.

**Figure 2:**
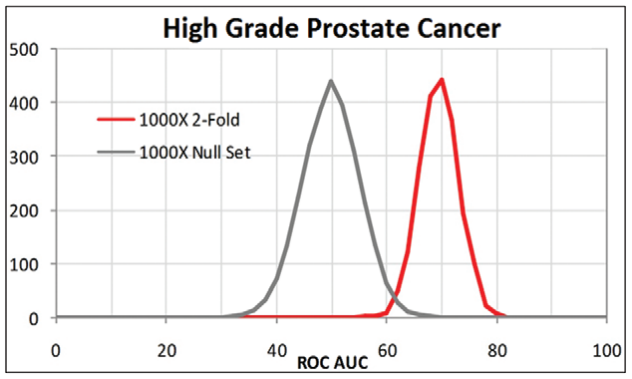
PCR data from a sample set of 122 PAXgene samples of prostate cancer from the G8 group and 138 PAXgene samples from the control group were performed in a 1000×2-fold cross-validation test. Histograms of AUC were plotted and compared; results showed AUCs from the PCR data were well separated from the null sets, with an overlap of less than 5%.

To estimate the possibility that the results were due merely to random chance, we performed two-fold cross-validations, in which half of the samples were used to define coefficients and thresholds for the model, which was then used to predict the remaining half of the samples. This process was iterated 1000 times using the actual data, first with the aggressive cancer status and a second time with the status randomly re-assigned. The distribution of the AUC ROC from each analysis resulted in two well-separated curves with less than 5% overlap from which we conclude that the observed performance is unlikely to be merely the result of random chance (Figure 2).

From the data, we built a logistic regression model combining PSA and gene expression ratios using G8 samples against controls to further enhance the discriminative ability of the panel. This achieved an AUC ROC of 0.99 on 69 G8 versus 101 controls of Cohort II (sensitivity  =  96%, specificity  =  100%).

This combined panel of PSA and gene expression ratios (see Table 3) defined from Cohort II was tested on Cohort III samples. All six duplexes remained significantly differentially expressed between G8 and controls (all p-values are ≤ .01). The equation built from the Cohort II study – with fixed parameters – was applied to the new Cohort III data set, to provide an independent evaluation of the differential performance of the biomarker panel. The AUC ROC was 0.88 on 54 G8 versus 57 controls (sensitivity  =  83%, specificity  =  98%).

**Table 3:** Combined PSA and mRNA model.

Constant	CRTAM	CXCR3	FCRL3	KIAA1143	KLF12	TMEM204	Log2PSA
−2.83	0.208	−0.729	0.752	−0.779	3.77	0.427	3.22

Inputs are CT values for genes and Log2 transformation of PSA in ng/ml.

All analyses reported above involved samples of controls or G8 cases and were used for model validation. However, the objective in this study was to estimate aggressiveness of the cancer. For this purpose we used Cohort IV, a set comprising cases of intermediate cancer grades (G6 n = 33, G7(3+4) n = 35, and G7(4+3) n = 43). For this analysis, control, G6 and G7(3+4) cases were considered non-aggressive cancers and G7(4+3) and G8 were considered aggressive cancers. ROC analysis (as applied for Cohorts I,II,III) is not appropriate here, as ROC calculation is best for simple positive and negative proportions within groups, and Cohort IV contained more complex subgroupings.

**Figure 3:**
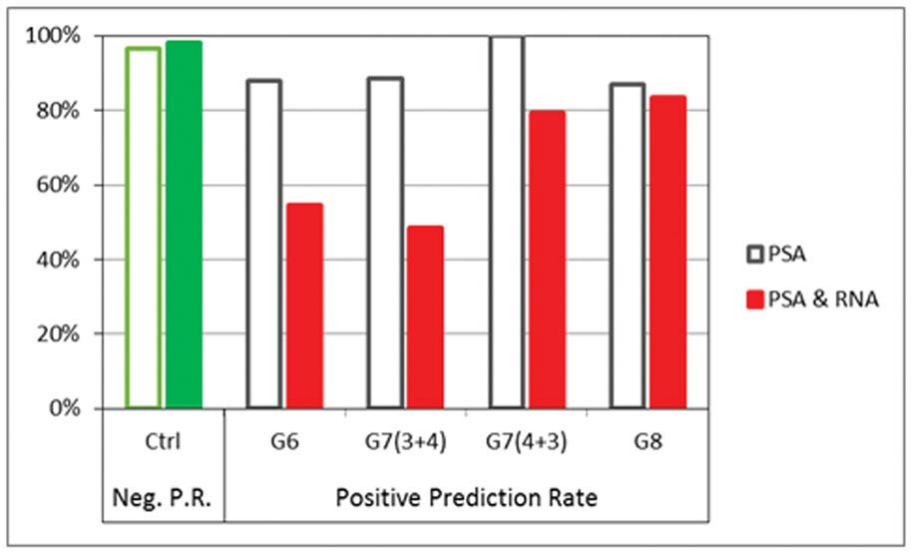
Predictions for independent Cohort III and Cohort IV samples. The negative prediction rate for control cases is charted along with the positive prediction rates for cancer cases. PSA alone has high positive predictive rates for all cancer grades (>87%) but the combined PSA and RNA panel has lower positive prediction rates for the less aggressive G6 and G7(3+4) subgroups, 55%, and 49% respectively) while nearly the same positive prediction rate for the more aggressive G7(4+3) as G8 groups (79% and 83% respectively.

Instead, for Cohort IV analysis, we evaluated the positive prediction rate for each subgroup, and found that 55% of G6, 49% of G7(3+4) and 79% of G7(4+3) were detected with the same signature that had identified the G8 aggressive cancer cases (Figure 3). By contrast, PSA level alone was unable to differentiate between the less aggressive G6, G7(3+4) and the more aggressive G7(4+3) groups, yielding positive prediction rates of 88%, 89% and 100%, respectively, in samples where PSA levels reached 4 ng/ml.

## Discussion

Our objective in this study was to identify blood-based biomarkers for aggressive prostate cancer. We collected samples at several locations, from both Asian and non-Asian subjects, in order to minimize ethnic and racial differences. Our strategy focused mainly on the identification of biomarkers for the highest-grade cancers (Gleason score 8–10), and we then applied the model to patients with Gleason score 7(4+3), Gleason score 7(3+4), Gleason score 6 and controls. The model was successful in distinguishing patients with high risk Gleason score 7(4+3) to Gleason score 10 from those with low to intermediate risk Gleason score 6 and Gleason score 7(3+4). The preliminary results of this seven gene model to predict aggressive prostate cancer are encouraging and need to be validated in a multi-site validation clinical study.

As confirmed in the results above, PSA on its own has a high positive prediction rate. The addition of the reported blood-based biomarker panel improved PSA accuracy for aggressive cancers. This was achieved by correcting for over-diagnosis of aggressive cancers in the G6 and G7 cohorts by about one-half (fewer cases should be expected to exhibit an aggressive cancer molecular signature in the lower-grade cancer cohorts).

We identified candidate biomarker genes and developed gene duplexes by combining an overexpressed gene with an underexpressed gene in order to amplify differential gene expression and normalize for individual variations (Table 2). Verification on independent Cohort III samples showed that we were successful in our efforts. This is represented by our analysis in control patients and confirmed Gleason score 8–10 prostate cancer patients, with a specificity of 80% or better. A gene-only multivariate predictive model built on Cohort II data was applied to Cohort III samples, and the specificity remained high at 83%.

The seven genes identified from blood-derived mRNA in this study are mainly involved in immune response, chemotaxis, and gene transcription regulation in carcinogenesis [22]–[25]. Of interest, our study found CRTAM significantly underexpressed in aggressive prostate cancer, suggesting a possible role for T-cell deficiency in prostate cancer. In addition, altered KLF expression has been found in tumors and tumor progression [26]–[29], and several investigations report that activator protein 2 alpha (AP-2alpha) plays an essential role in tumorigenesis [30], [31].

Many men with early prostate cancer will never progress to late stage cancer. The subset of men with indolent disease would be excellent candidates for active surveillance. However, there is a lack of clear criteria to differentiate between those most at risk for aggressive cancer and those whose disease will follow an indolent course [4], [32], [33]. PSA as a lone indicative biomarker has a high false positive and significant false negative rate [34]. It exposes many men to repeated unnecessary biopsies, with risks of pain, infection, sepsis, and potential cascading downstream consequences, such as radical prostatectomy with side-effects of impotence and incontinence [35]. Thus an important clinical challenge in prostate oncology is to identify, within the population of PSA-positive men, those with high-grade or aggressive cancer, without requiring all patients to undergo a painful tissue biopsy.

The blood-based biomarker signature reported here identifies prostate cancers with Gleason scores between 7(4+3) and 10. Replicated in a more generalized and representative population, this biomarker signature can be refined and used to form the basis of a simple blood test. Used in conjunction with PSA as a risk stratification tool, the reported signature can identify men at risk of having high-grade prostate cancer. Biopsy, saturation biopsy, confirmation and intervention can be recommended for men of this category, as treatment for higher-grade cancer has been shown to positively affect 5-year survival rates as compared with observation [36]. Conversely, men without this biomarker signature may have more confidence in choosing “active surveillance” over immediate therapy.

## Supporting Information

Appendix S1 **Details of biopsies of prostate cancer samples.**
(DOC)

## Acknowledgments

We would like to acknowledge the contributions of Dr G Lee and Dr WL Chong. The authors gratefully acknowledge the editorial assistance of Isolde Prince and David J Novak.
