# Supplementary material for: Blood-Based Biomarkers of Aggressive Prostate Cancer
Source: PLoS One. 2012 Sep 28;7(9):e45802. doi: 10.1371/journal.pone.0045802 (PMC3461021; doi:10.1371/journal.pone.0045802)
Supplement: Appendix S1 — Details of biopsies of prostate cancer samples. (DOC) [file pone.0045802.s001.doc]

**Appendix S1**

**Details of Biopsies of Prostate Cancer Samples**

Cohort II: G8

| Procedure | Number of samples |
| --- | --- |
| RPP | 16 |
| TRUS (transrectal ultrasound) | 43 |
| TURP (transurethral resection of prostate) | 21 |
| Total | 80 |

Cohort III: G8

| Procedure | Number of samples |
| --- | --- |
| RPP | 12 |
| TRUS (transrectal ultrasound) | 27 |
| TURP (transurethral resection of prostate) | 16 |
| Total | 54 |

Cohort III: G7 (3+4)

| Procedure | Number of samples |
| --- | --- |
| RPP | 16 |
| TRUS (transrectal ultrasound) | 12 |
| TURP (transurethral resection of prostate) | 7 |
| Total | 35 |

Cohort III: G7 (4+3)

| Procedure | Number of samples |
| --- | --- |
| RPP | 20 |
| TRUS (transrectal ultrasound) | 15 |
| TURP (transurethral resection of prostate) | 8 |
| Total | 43 |

Cohort III: G6

| Procedure | Number of samples |
| --- | --- |
| RPP | 12 |
| TRUS (transrectal ultrasound) | 15 |
| TURP (transurethral resection of prostate) | 6 |
| Total | 33 |
